# Effect of Chinese Herbal Medicine Mixture 919 Syrup on Regulation of the Ghrelin Pathway and Intestinal Microbiota in Rats With Non-alcoholic Fatty Liver Disease

**DOI:** 10.3389/fmicb.2021.793854

**Published:** 2021-12-24

**Authors:** Manman Chen, Jingwei Xing, Danqing Pan, Pengfei Gao

**Affiliations:** ^1^Department of Integrative Medicine, Huashan Hospital, Fudan University, Shanghai, China; ^2^Department of Traditional Chinese Medicine, Yueyang Hospital of Integrated Traditional Chinese and Western Medicine, Shanghai University of Traditional Chinese Medicine, Shanghai, China; ^3^Department of Traditional Chinese Medicine, Jinshan Hospital, Fudan University, Shanghai, China

**Keywords:** 919 syrup, non-alcoholic fatty liver disease – NAFLD, appetite, Ghrelin pathway, intestinal microbiota

## Abstract

As a manifestation of metabolic syndrome in the liver, non-alcoholic fatty liver disease (NAFLD) has become the top cause of liver disease in many countries. Recent studies have shown that intestinal microbiota disorder plays an important role in the occurrence and development of NAFLD and that regulating intestinal microbiota provides a new option for NAFLD treatment. In addition, research indicates that risk of NAFLD increases as body mass index rises, and interventions that reduce body weight and change diet can help to lower the incidence of NAFLD. Studies have found that 919 syrup may effectively treat NAFLD in rats by improving liver function and lipid metabolism and regulating body weight and feed intake, however, its potential toxicity and the specific mechanism by which it controls this disease require further exploration. This study assesses both the toxicity of 919 syrup and its regulatory effect on the appetite-related Ghrelin pathway and intestinal microbiota of rats with NAFLD. Results indicate that 919 syrup has no obvious side-effects on body weight, feed intake, blood glucose level, hepatorenal function, and liver tissue structure of normal rats. Moreover, 919 syrup can reverse abnormal changes to expression of Ghrelin pathway genes related to appetite in both the brain and stomach and repair alterations to the intestinal microbiota in rats with NAFLD. This herbal medicine is a safe and promising therapeutic drug for the treatment of NAFLD.

## Introduction

As a result of the worldwide increase in obesity, the manifestation of metabolic syndrome in the liver, non-alcoholic fatty liver disease (NAFLD), is now a primary cause of chronic liver disease in many countries. The incidence of NAFLD in patients with metabolic syndromes, including type 2 diabetes, hyperlipidemia, obesity and hypertension, is significantly higher than in the general population. NAFLD includes simple liver steatosis and non-alcoholic steatohepatitis. Progression of non-alcoholic steatohepatitis can cause more severe liver cirrhosis and hepatocellular carcinoma. The main pathological impact of NAFLD is the accumulation of fat in the liver, however, the specific mechanism by which this occurs is not yet clear. The “second hit” theory, liver steatosis caused by insulin resistance along with pathological changes like inflammation and fibrosis of liver tissue caused by oxidative stress and other factors, is widely accepted (Day and James, 1998). NAFLD poses a serious threat to human health and requires focus on the development of effective treatments.

919 syrup (919TJ), Chinese drug invention patent, CN107929413B, has been used in scientific and clinical research for more than 30 years and has been shown to protect liver cells by improving liver function, repairing liver tissue (Huang et al., 1995), increasing anti-viral response (Huang and Gao, 2002), decreasing fibrosis (Huang et al., 2003), and reducing hepatocyte steatosis (Chen et al., 2020). Findings indicate that 919 syrup is safe to use (Gao and Zhang, 2005) but no formal toxicity testing has been carried out.

Many studies (Xing et al., 2013; Li et al., 2017) have shown that body weight and feed intake are closely related to the development of NAFLD. The incidence of NAFLD increases as body mass index (BMI) rises, and interventions targeting body weight and feed intake can improve lipid metabolism and reduce the incidence of NAFLD. Importantly, both feed intake and body weight decrease in NAFLD rats that are treated with 919 syrup (Chen et al., 2020). Ghrelin is a growth hormone releasing peptide found in the stomachs of mice and humans. Acylated Ghrelin is an endogenous ligand of growth hormone secretagogue receptor (GHS-R) that promotes release of growth hormone, increases appetite, and regulates digestive system function and energy metabolism (Jiao et al., 2017). In this study, the specific mechanism of 919 syrup on body weight and feed intake in rats with NAFLD was studied by assessing changes to the appetite-related Ghrelin pathway.

The stability of intestinal microbiota allows it to resist invasion of pathogenic bacteria, develop effective immune responses, uptake and absorb host energy, and manage fat, and is thus critical to human health (Raman et al., 2005; Tilg, 2010; Del Chierico et al., 2012). Imbalance of the intestinal microbiota is associated with the development of serious metabolic-related diseases. In recent years, more attention has focused on the role of intestinal microbiota in the development of NAFLD. The imbalance of intestinal microbiota impacts metabolism by increasing liver inflammation, destroying the intestinal mucosal barrier, affecting energy metabolism, and producing a large number of toxic metabolites (Chassaing et al., 2014). Regulating intestinal microbiota has become a new avenue for treatment of non-alcoholic fatty liver.

Experiments performed in this study were divided into three parts. The first assessed the toxicity of 919 syrup. The second studied the mechanism of 919 syrup on the weight and feed intake of NAFLD rats by assessing Ghrelin pathway gene expression. The third investigated whether 919 syrup regulated the intestinal microbiota in rats in NAFLD, and the potential correlation between changes in intestinal dominant microbiota and Ghrelin pathway gene expression. This study explored the therapeutic mechanism of 919 syrup on NAFLD from different perspectives, in order to prove the effectiveness and safety of 919 syrup in the treatment of NAFLD.

## Materials and Methods

### Experimental Animals

Fifty specific pathogen-free (SPF) male Sprague–Dawley (SD) rats weighing 150 ± 10 g, were provided by Shanghai Xipuer-Bikai Laboratory Animal Co., Ltd. (SCXK, Shanghai, China). The experimental animals were housed in the animal laboratory building of the Shanghai Public Health Clinical Center in Shanghai, China. The animal room was kept at 20–22°C and alternated between light and dark every 12 h. The animal experiment protocol was approved by the Animal Care and Use Committee of Fudan University Public Health Clinical Center and followed the ethical review guidelines for laboratory animal welfare.

### Establishment of Non-alcoholic Fatty Liver Disease Rat Model

Sprague–Dawley male rats in the NAFLD and 919TJ groups were fed a high-fat diet consisting of 31.1% calories from fat, 53.3% calories from carbohydrates, 77% basal diet, 10% lard, 2.5% cholesterol, 0.5% chocolate, 5% egg yolk powder, and 5% sucrose. After consuming this diet for 4 weeks, fatty degeneration of the liver tissue was assessed by hematoxylin-eosin (HE) staining of the liver.

### Preparation of 919 Syrup

919 syrup is composed of over 10 Chinese herbs including kiwifruit (20 g), salvia (10 g), atractylodes (5 g), schisandra (5 g), orange peel (2.5 g), and Bupleurum (2 g). 919TJ was produced by the pharmaceutical factory of the Shaanxi University of Chinese Medicine (Xi’an, Shaanxi, China) using the formula provided by our research group, and the final drug concentration was 1.35 g/mL. Based on conversion of the clinical dose of 919 syrup along with human and animal body surface areas, different doses of 919 TJ (20 ml/kg, 10 ml/kg, 5 ml/kg) were used in prior experiments (Huang et al., 1995; Huang and Gao, 2002; Xu et al., 2017; Chen et al., 2020), with 10 ml/kg showing good efficacy. The concentration used for administration was set at 13 g/kg/d for this study, and the dose of intragastric administration was 10 ml/kg/d.

### Experimental Design

#### Toxicity Test of the 919 Syrup

**FIGURE 1:**
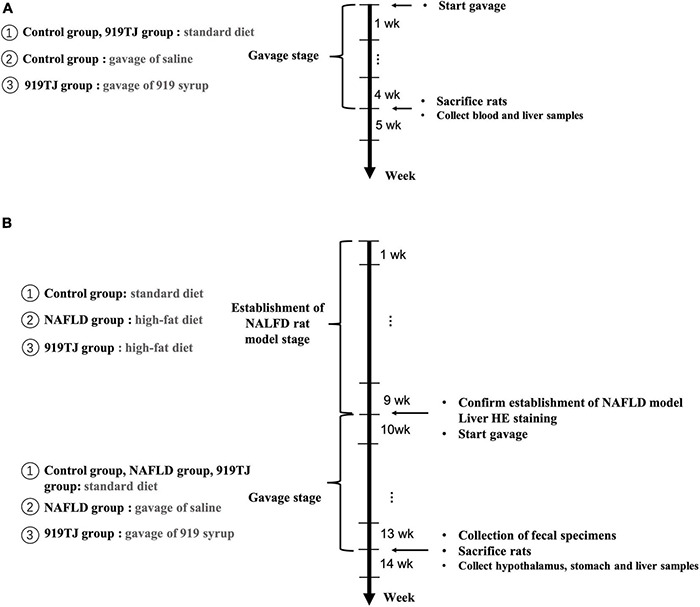
Timeline of 919 syrup’s toxicity test **(A)**. Timeline of experiment assessing effect of 919 syrup on NAFLD rats **(B)**.

Twenty male SD rats were randomly divided into the control and 919TJ groups (*n* = 10 per group). Both groups received a standard diet each day, and while rats in the 919TJ group were fed 919 syrup daily, rats in the control group were given the same volume of saline daily. Both groups of rats were fed continuously for 4 weeks and daily body weight and feed intake were recorded. At the beginning of the fifth week, the rats were sacrificed, blood and liver samples collected, serum biochemical indexes, blood glucose, and hepatorenal function were detected, and liver histological changes were observed by HE staining (Figure 1A).

#### Effects of 919 Syrup on Appetite-Related Brain-Stomach Ghrelin Pathway in Non-alcoholic Fatty Liver Disease Rats

Thirty male SD rats were randomly divided into control, NAFLD and 919TJ groups (*n* = 10 per group). The control group received the standard diet, while the NAFLD and 919TJ groups were fed the high fat diet for 9 weeks to establish NAFLD. Liver HE staining confirmed that disease was established. From the beginning of the tenth week, rats in the 919TJ group were given 919 syrup by gavage, and rats in NAFLD group were given the same volume of normal saline by gavage. This method of feeding continued for 4 weeks and body weight and feed intake were recorded daily. At the beginning of week 14, fecal samples were collected for the experiment described in section “Regulatory Effect of 919 Syrup on Intestinal Microbiota in Rats With NAFLD” and the rats were sacrificed. Hypothalamus, stomach and liver samples were collected to detect the expression of genes and proteins associated with the brain-stomach Ghrelin pathway (Figure 1B).

#### Regulatory Effect of 919 Syrup on Intestinal Microbiota in Rats With Non-alcoholic Fatty Liver Disease

As described in section “Effects of 919 Syrup on Appetite-Related Brain-Stomach Ghrelin Pathway in Non-alcoholic Fatty Liver Disease Rats,” rats were fed until the beginning of week 14 and fecal samples were collected from rats in each group prior to sacrifice. All samples were immediately stored in sterile containers and frozen at –80°C. Fecal samples were removed to extract genomic DNA. 16S rDNA was amplified by polymerase chain reaction (PCR) and sequenced by Illumina Miseq to obtain the sequence of template DNA fragments. Bioinformatics analysis was performed on the Miseq sequencing data (Figure 1B).

### Quantification of mRNA Expression

5-Ht_2c_ Receptor (*5-Ht_2c_R*), 5-Ht_2b_ Receptor (*5-Ht_2b_R*), *Leptin*, Agouti-related protein (*Agrp*), Neuropeptide Y (*Npy*), Corticotrophin-releasing factor (*Crf*), Proopiomelanocortin *(Pomc*), *Ghrelin*, and *GHSR* mRNA levels in the hypothalamus and stomach were determined by quantitative real time PCR (qRT-PCR). RNEasy Mini Kit was used to isolate total RNA from tissue samples, and the complementary DNA was synthesized using IMPROM-IITM reverse transcription system. The qRT-PCR analysis was performed using primers listed in Supplementary Table S1 and the Quantifast SYBR Green RT-PCR kit. The experiment was repeated three times on each sample. Using the iCycler iQ™ fluorescent q-RT-PCR system (Bio-Rad Laboratories), thermal cycles were performed at 95°C for 5 min, followed by 40 cycles at 95°C for 10 s and 60°C for 30 s. After standardization based on GAPDH transcription, relative mRNA expression of each gene was measured using the 2^–ΔΔCt^ method.

### Western Blot

Rat liver tissue was lysed in RIPA buffer and the supernatant was removed after centrifugation. Total protein concentration was determined using the BCA protein assay kit (Thermo, United States), with equivalent protein samples separated by 12% SDS-PAGE and electrically transferred to a polyvinylidene fluoride (PVDF) membrane (Millipore, United States). The PVDF membrane was placed in 5% skimmed milk powder and blocked for 1 h. The milk powder was washed with TBST, and the membrane was incubated overnight with primary anti-Ghrelin and anti-GAPDH antibodies (CST, United States) at 4°C. The PVDF membrane was then washed with TBST, incubated with the peroxidase-labeled secondary antibody on a shaker for 1 h at room temperature, and cleaned with TBS. The signal was measured with an enhanced luminescent substrate. Each of the three groups had six immunoreactive bands which were quantified with ImageJ (National Institutes of Health, United States).

### Hematoxylin-Eosin Staining of Liver

Tissue blocks from the right lobe of rat liver were collected and immediately fixed in 10% formaldehyde for 48 h. Animal tissues were sliced into 0.2–0.5 cm slices, and dehydrated in 70% alcohol for 2 h, 80% alcohol for 2 h, 95% alcohol (I, II 2 h), and waterless alcohol (I, II 1 h), and made transparent with xylene (I, II 1 h each). The dehydrated transparent tissues were soaked in melted paraffin wax, placed in the encapsulating device, dripped into the liquefied wax for tissue burial, and placed on ice to be solidified. The wax cubes were fixed on the slicer, cut into 5-μm-thick slices, spread on slides coated with anti-stripping agent, and baked in an oven at 65°C for 3 h. The paraffin sections were dyed in hematoxylin for 5 min, washed with distilled water, differentiated with 1% diluted hydrochloric acid, fully rinsed in running water to turn blue for about 20 min, stained with eosin for 5 min, and rinsed with running water. Dyed slices were dehydrated with gradient alcohol (70, 80, 95, and 100% for 5 min each), and made transparent with xylene (I, II for 5 min each). Finally, slides were dripped with neutral glue and sealed with the cover slide.

### 16S qPCR Quantification of Bacterial DNA

16S rDNA sequencing was performed to classify bacterial DNA that was isolated from the fecal microbiome. Genomic DNA was extracted from the fecal specimen using 1% agar glycogel electrophoresis. The V3-V4 region of bacterial 16S rDNA was amplified by qPCR. The forward primer (338F) was 5′-ACTCCTACGGGAGGCAGCAG-3′ and the reverse primer (806R) was 5′-GGACTACHVGGGTWTCTAAT-3′. Three repetitions were performed per sample. The qPCR products were recovered using AxyPrepDNA gel recycling kits (AXYGEN, United States), eluted by Tris-HCl buffer, and tested by 2% agarose gel electrophoresis. The qPCR products were detected and quantified using the QuantiFluor*™*-ST blue fluorescence quantification system (Promega, United States). Sequencing was performed on the Illumina Miseq platform, and fluorescent signal emissions were statistically analyzed to obtain the sequence of the template DNA fragment.

### Bioinformation Analysis

The paired-end reads obtained by Miseq sequencing were spliced according to the overlap relationship, and the sequence was quality controlled and filtered. Operational taxonomic units (OTU) cluster analysis and species taxonomy analysis were performed after the sequences were determined. Based on results of OTU cluster analysis, OTU was analyzed with various diversity indexes and using taxonomic information, statistical analysis of the community structure was carried out at various classification levels. Data at the phylum and genus level were assessed in this study.

Bioinformatics analysis was performed on the Miseq sequencing data, including intestinal microbiota richness and diversity index (Rank-Abundance curves), Alpha diversity analysis (Sobs, Shannon, Simpson, etc.), β-beta diversity analysis (Hierarchical clustering, PCA and PCoA), species composition analysis (Heatmap, Barplot), relationship between samples and species (Circos), and the correlation between the dominant intestinal microbiota and genes related to the Ghrelin pathway (Spearman correlation heatmap).

### Statistical Analysis

SPSS (Version 22.0. Chicago, SPSS Inc.) was used for statistical analysis, except for assessment of intestinal microbiota, and the data was expressed as mean ± standard deviation (x ± s). The chart was drawn using PRISM8. One-way analysis of variance (ANOVA) was used to evaluate the differences between groups. *P < 0.05, ^**^P < 0.01, ^***^P < 0.001. *P* < 0.05 was considered statistically significant.

## Results

### Toxicity Test of the 919 Syrup

**FIGURE 2:**
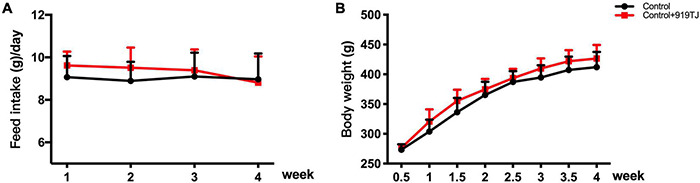
Effect of 919 syrup on feed intake and body weight in normal rats. **(A)** Feed intake. **(B)** Body weight.

Figure 2 shows changes in body weight and feed intake in the two groups of rats over 4 weeks. The rats in both groups were fed a normal diet, and the control + 919TJ group was given 919 syrup (10 ml/kg/d) daily. After 4 weeks, there was no statistical difference in body weight and feed intake between the control and control + 919TJ groups (*P* > 0.05). These results showed that 919 syrup did not negatively impact the body weight and feed intake of normal rats.

**FIGURE 3:**
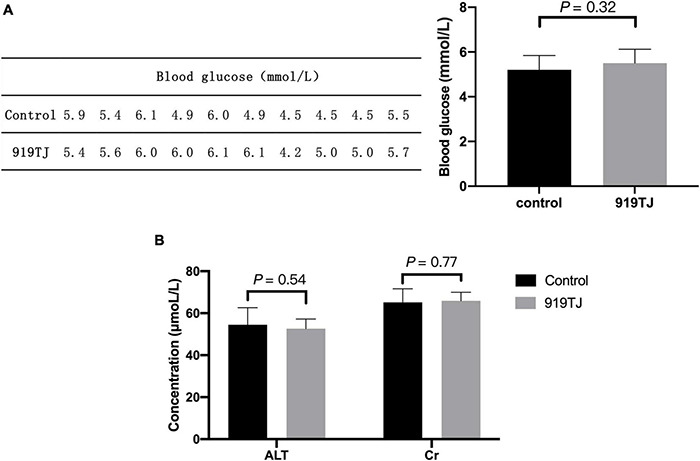
Effect of 919 syrup on blood glucose and hepatorenal function in normal rats. **(A)** Glucose. **(B)** Hepatorenal function.

Figure 3A shows the blood glucose levels in two groups of rats (*n* = 10 per group) after 4 weeks of feeding. The blood glucose of rats in the control and 919TJ groups were 5.2 ± 0.64 mmol/L and 5.5 ± 0.62 mmol/L, respectively, and there was no statistical difference between the two (*P* > 0.05). These results showed that 919 syrup did not negatively impact the blood glucose of normal rats.

Figure 3B shows changes in hepatorenal function indicators (ALT, Cr) after 4 weeks of feeding. The ALT value of the control and 919TJ groups were 54.5 ± 8.2 μmol/L and 52.6 ± 4.6 μmol/L, respectively, and there was no statistical difference between the two groups (*P* > 0.05). The Cr value of rats in the control and 919TJ groups was 65.2 ± 6.5 μmol/L and 65.9 ± 4.1 μmol/L, respectively, and there was no significant difference between the two. These results showed that 919 syrup had no obvious negative impact on the hepatorenal function of normal rats.

**FIGURE 4:**
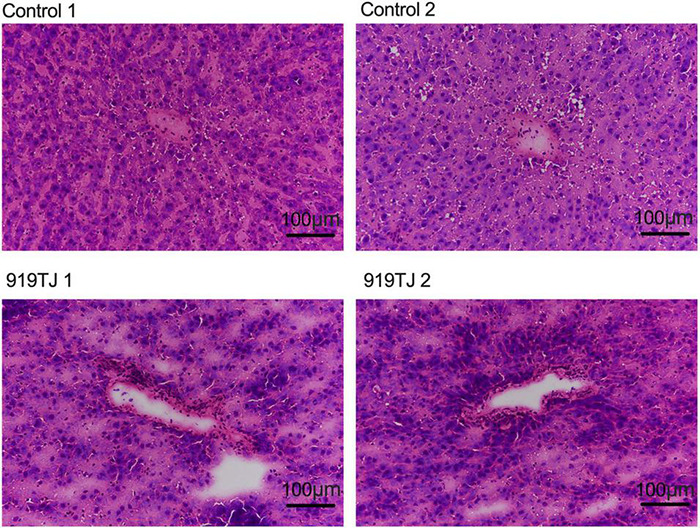
HE staining of liver in the control and 919TJ group.

Figure 4 shows HE staining images of rat livers in both groups after 4 weeks of feeding. HE images of the rat livers from both groups showed that the liver cells were normal in shape, orderly in arrangement, and radially arranged from the center of the central vein. These results showed that 919 syrup did not negatively impact the liver tissue structure of normal rats.

### Effect of 919 Syrup on the Appetite-Related Ghrelin Pathway in Non-alcoholic Fatty Liver Disease Rats

**FIGURE 5:**
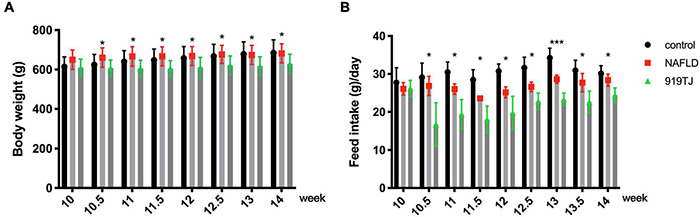
Effect of 919 syrup on body weight and feed intake in rats with NAFLD. **(A)** Body weight. **(B)** Feed intake. *P < 0.05, ***P < 0.001 (NAFLD group vs. 919TJ group, *n* = 8 per group).

From the beginning of the 10th week, rats in the 919TJ group were given 919 syrup by gavage, and rats in the NAFLD group were given the same volume of normal saline by gavage. Body weight and feed intake were recorded during gavage. As shown in Figure 5, compared with the rats in the NAFLD group, the weight and feed intake of the rats in the 919TJ group decreased after feeding with 919 syrup during the gavage period (*P < 0.05, ^***^P < 0.001).

**FIGURE 6:**
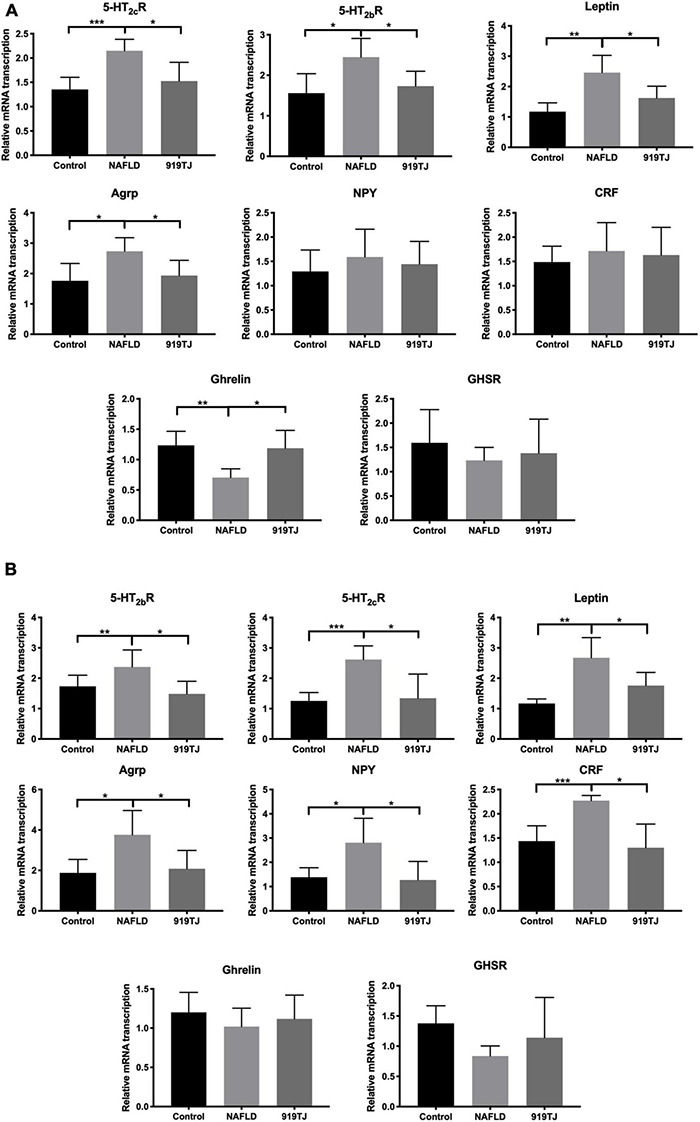
Effect of 919 syrup on Ghrelin pathway-related genes in the hypothalamus and stomach of NAFLD rats. **(A)** Hypothalamus. **(B)** Stomach. *P < 0.05, **P < 0.01, ***P < 0.001 (*n* = 8 per group).

Figures 6A,B show changes in expression of Ghrelin pathway-related genes in the hypothalamus and stomach of the three groups of rats. As compared to the control group, *5-HT_2c_R, 5-HT_2b_R, Leptin, AGRP, NPY*, and *CRF* expression increased in the hypothalamus and stomach of NAFLD rats, while *Ghrelin* and *GHSR* expression decreased in both sites. As compared to the NAFLD group, *5-HT_2c_R, 5-HT_2b_R, Leptin, AGRP, NPY*, and *CRF* expression decreased in the hypothalamus and stomach of the 919TJ group, while *Ghrelin* and *GHSR* expression increased in both sites (*P < 0.05, ^**^P < 0.01, ^***^P < 0.001). These results showed that 919 syrup can reverse the abnormal expression of Ghrelin pathway-related genes in the hypothalamus and stomach of NAFLD rats.

**FIGURE 7:**
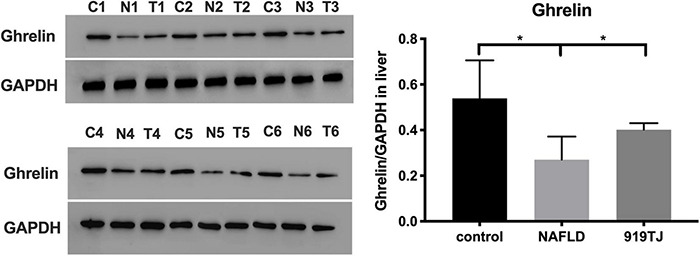
Changes in Ghrelin protein levels in the liver of rats as shown by western blot. C, control group. N, NAFLD group. T, 919TJ group. *P < 0.05 (*n* = 6 per group).

Figure 7 shows Ghrelin protein levels in the liver of rats in the three groups. As compared to the control group, Ghrelin protein levels decreased in the liver of rats in the NAFLD group (*P* < 0.05). As compared to the NAFLD group, Ghrelin protein levels increased in the liver of rats in the 919TJ group (*P* < 0.05). Results showed that 919 syrup can recover Ghrelin protein levels in the liver of rats with NAFLD.

### Effect of 919 Syrup on Intestinal Microbiota in Rats With Non-alcoholic Fatty Liver Disease (Gut Microbiota Analysis)

#### Richness and Diversity Index

**FIGURE 8:**
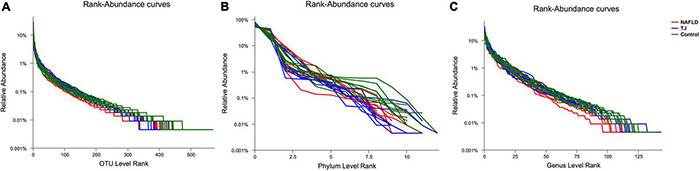
Rank-Abundance curve of intestinal microbiota. The abscissa is the rank of species’ number (or OTU) at a taxonomic level, and the ordinate is the relative percentage of species’ number at that taxonomic level. **(A)** OUT level. **(B)** Phylum level. **(C)** Genus level. TJ: 919 syrup group.

Illumina MiSeq platform for high-throughput pyrosequencing of fecal genes from rats was used to assess the effect of 919 syrup on gut microbiota. Species diversity was analyzed using the rank abundance curve, which indicates species richness and community evenness (Figure 8). Species richness is reflected by the width of the curve, and the larger the range of the curve on the horizontal axis, the higher the species richness. As shown in the Figure 8, the width of curve was larger for the control group than the NAFLD group at the level of OUT, Phylum and Genus, and the curve width was similar between the 919TJ and control groups, indicating that species richness was higher in the 919TJ group than the NAFLD group and more similar to the control group. The shape of the curve reflects species diversity. Smoother curves indicate higher species diversity, while rapid and steep declines indicate that the dominant microbiota accounts for a high proportion of the sample and diversity is low. The curve of the 919TJ and control groups decreased more gently than the curve of the NAFLD group. These findings indicate that the 919TJ group had higher species diversity than the NAFLD group, and was more similar to the species diversity observed in the control group.

#### Alpha Diversity Analysis

**FIGURE 9:**
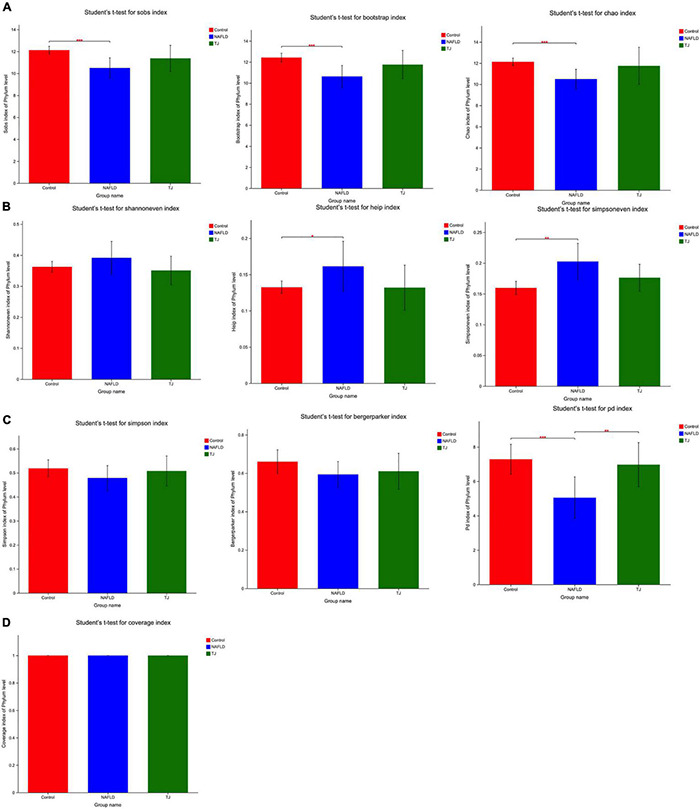
Alpha diversity analysis of intestinal microbiota (phylum level). **(A)** Community richness. **(B)** Community evenness. **(C)** Community diversity. **(D)** Community coverage. *P < 0.05; **P < 0.01; ***P < 0.001.

As shown in Figure 9A, community richness was reflected by sobs, bootstrap and chao. NAFLD rat intestinal microbiota had lower community richness than the control group, and the difference was statistically significant (^***^P < 0.001). In contrast, intestinal microbiota in the 919TJ group showed higher community richness. These results showed that 919 syrup reversed the decline in intestinal microbial richness under the NAFLD conditions, such that the intestinal microbial richness of rats in the 919TJ group was more like that observed in the control group.

As shown in Figure 9B, community evenness was reflected by simpsoneven, shannoneven, and heip. Community evenness was higher in NAFLD rat intestinal microbiota than controls and the difference was statistically significant (*P < 0.05; ^**^P < 0.01). In contrast, community evenness in 919TJ intestinal microbiota was decreased. Results showed that 919TJ reversed the increase of intestinal microbial evenness under NAFLD conditions, such that the intestinal microbial evenness of rats in the 919TJ group was more like that observed in the control group.

As shown in Figure 9C, community diversity was reflected by simpson, berger parker, and PD. Community diversity was lower in NAFLD rat intestinal microbiota than controls and the difference was statistically significant (^***^P < 0.001). In contrast, the community diversity was higher in rat intestinal microbiota in the 919TJ group (^**^P < 0.01). Results showed that 919 syrup reversed the decline of intestinal microbial diversity under NAFLD conditions, and made the intestinal microbial diversity of rats in 919TJ group closer to that seen in the control group.

As shown in Figure 9D, community coverage refers to the coverage of each sample library. The higher the value, the higher the probability that the sequence in the sample will be measured. The values of the three groups of coverage were all one, indicating that the sequencing results represented the real conditions of the microorganisms in each sample.

#### Species Composition Analysis

**FIGURE 10:**
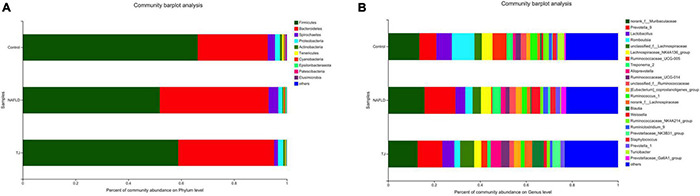
Community barplot analysis of intestinal microbiota. **(A)** Phylum level (Others: <0.001). **(B)** Genus level (Others: <0.02). The ordinate is the sample name, the abscissa is the proportion of species in the sample, columns with different colors represent different species, and the length of the columns represents the proportion of the species.

**FIGURE 11:**
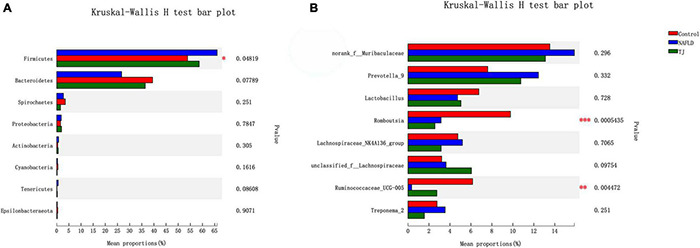
Kruskal–Wallis *H* test bar plot of intestinal microbiota. **(A)** Phylum level. **(B)** Genus level. The *Y* axis represents the bacterial names at the phylum/genus level, the *X* axis represents the average relative abundance of bacteria in different groups, the columns with different colors represent different groups, and the rightmost number is the *P*-value. *P ≤ 0.05, ^**^P ≤ 0.01, ^***^P ≤ 0.001.

Community barplot analysis (Figure 10), Kruskal–Wallis H test bar plot (Figure 11), heatmap (Supplementary Figure S1), and Circos map (Supplementary Figure S2) help to elucidate which bacteria are dominant in the three groups of rat intestinal microbiota at the phylum and genus level, and the proportion of each dominant bacteria. At the phylum level, the composition of the intestinal microbiota was similar between the three groups, and the primary bacteria were *Firmicutes*, *Bacteroidetes*, *Spirochaetes*, *Proteobacteria*, *Actinobacteria*, *Tenericutes*, *Cyanobacteria*, and *Epsilonbacteraeota*. The proportion of bacteria observed in NAFLD rat intestinal microbiota was different than those observed in the control group with decreased ratios of *Firmicutes*, *Proteobacteria*, *Actinobacteria*, and *Tenericutes*, and increased ratios of *Bacteroidetes*, *Spirochaetes*, *Cyanobacteria*, and *Epsilonbacteraeota*. In contrast to the NAFLD group, *Firmicutes*, *Proteobacteria*, *Actinobacteria*, and *Tenericutes* proportions were higher in the intestinal microbiota of rats in the 919TJ group, and *Bacteroidetes, Spirochaetes, Cyanobacteria*, and *Epsilonbacteraeota* proportions were lower. The similarity in intestinal microbiota observed between the 919TJ and control groups indicates that 919 syrup reversed the abnormal bacterial composition in NAFLD rat intestinal microbiota at the phylum level.

At the genus level, the composition of the intestinal microbiota was similar between the three groups, and the primary bacteria were *norank_f_Muribaculaceae, Prevotella_9, Lactobacillus, Romboutsia, unclassified_f_Lachnospiraceae, Lachnospiraceae_NK4A136_group, Ruminococcaceae_UCG-005, Treponevotella_2, Alloprevotella, Ruminococcaceae_UCG-014*, and *unclassified_f_Ruminococcaceae*. The composition of the intestinal microbiota of NAFLD rats differed from the control group, with increased ratios of *norank_f_Muribaculaceae, Prevotella, unclassified_f_Lachnospiraceae, Treponema_2, Alloprevotella* and *unclassified_f_Ruminococcaceae*, and decreased ratios of *Lactobacillus, Romboutsia, Lachnospiraceae_NK4A136_group, Ruminococcaceae_UCG-005* and *Ruminococcaceae_UCG-014*. In contrast to NAFLD group, the proportions of *Lactobacillus, unclassified_f_ Lachnospiraceae, Ruminococcaceae_UCG-005, Alloprevotella* and *Ruminococcaceae_UCG-014* were higher in the intestinal microbiota of rats in the 919TJ group and the proportions of *norank_f_Muribaculaceae, Prevotella, Romboutsia, Lachnospiraceae_NK4A136_group, Treponema_2, unclassified_f_Ruminococcaceae* were lower. The similar composition of intestinal microbiota in the 919TJ and control groups show that 919 syrup reversed the abnormal composition of NAFLD rat intestinal microbiota at the genus level.

#### β Diversity Analysis

β diversity analysis at the phylum level using hierarchical clustering, PCA, and PCOA was used to demonstrate similarities or differences between groups.

To study the similarity or difference relationship of the community structure of different samples, the arithmetic mean unweighted pair-wise clustering method was used for hierarchical clustering, and the tree graph was constructed at the phylum level (Supplementary Figure S3). The distance between branches indicates the difference in species composition between samples. This analysis showed that, at the phylum level, sample branches of the NAFLD group were concentrated at the top, and that the distance between the branches of the NAFLD group was far from both the control and 919TJ groups. In contrast, the distance between branches of the control and 919TJ groups were closer than either was to the NAFLD group, indicating that the difference in community composition between the control and 919TJ groups was smaller than that observed between the control and NAFLD groups.

In this study, multivariate analysis methods like principal component analysis (PCA) based on Euclidean distance and principal coordinate analysis (PCOA) based on Unifrac distance were used to analyze overall structural changes of intestinal microbiota.

**FIGURE 12:**
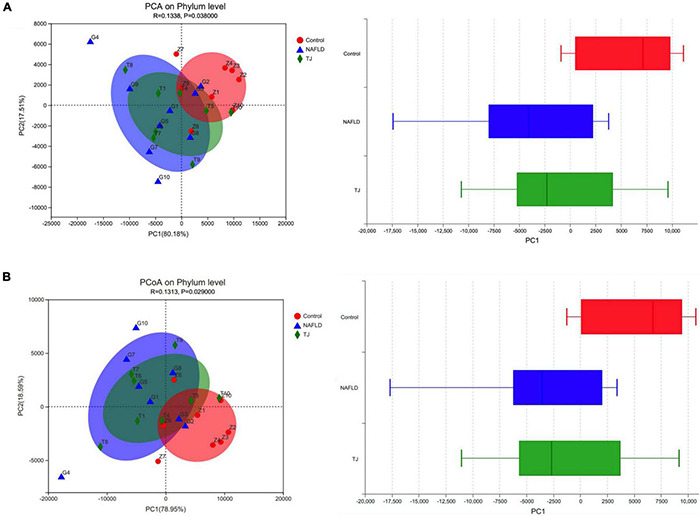
PCA and PCOA analyses of intestinal microbiota. The *X* and *Y* axes represent two selected principal component axes. Percentage represents the degree of explanation of the principal component to the difference in sample composition. The scales on the *X* and *Y* axes are relative distances. The closer the two sample points, the more similar the species composition of the two samples. The boxplot represents the distribution dispersion of the three groups of samples on the PC1 axis. **(A)** PCA. **(B)** PCOA. Z, control group. G, NAFLD group. T, 919TJ group.

At the phylum level (Figure 12), the symbols of samples in the NAFLD group were far from samples in the control group, while the symbols from samples in the 919TJ group were closer to the control than NAFLD groups. The NAFLD group clustered away from other groups while the 919TJ and control groups were close, suggesting that the bacterial community observed in the 919TJ group was similar to that observed in the control group. Using PCA and PCOA results, a boxplot was made for different grouped samples on the first principal component axis to visually present the discretization of different grouped samples. Median values of the samples from different groups were close, indicating that sample composition was similar. Compared with the NAFLD group, the median value of the 919TJ group was closer to that observed in the control group, indicating that the bacterial community composition of the 919TJ group was closer to that observed in the control group than the NAFLD group.

β diversity analysis revealed significant differences in the composition of the intestinal microbiota of NAFLD rats than normal rats, while the intestinal microbial community composition of NAFLD rats treated with 919 syrup was closer to that seen in normal rats.

#### Correlation Analysis of Intestinal Dominant Microbiota and Ghrelin Pathway

Spearman correlation analysis was used to explore the relationship between intestinal microbiota and the Ghrelin pathway in rats. Correlation analysis between the three groups of rats’ dominant intestinal microbiota and Ghrelin pathway-related molecules was performed, and heat maps were used to represent the results. The heatmap used correlation values to visualize the relationship between different bacteria in the samples and environmental variables and assess the correlation between microbial classification and environmental variables.

**FIGURE 13:**
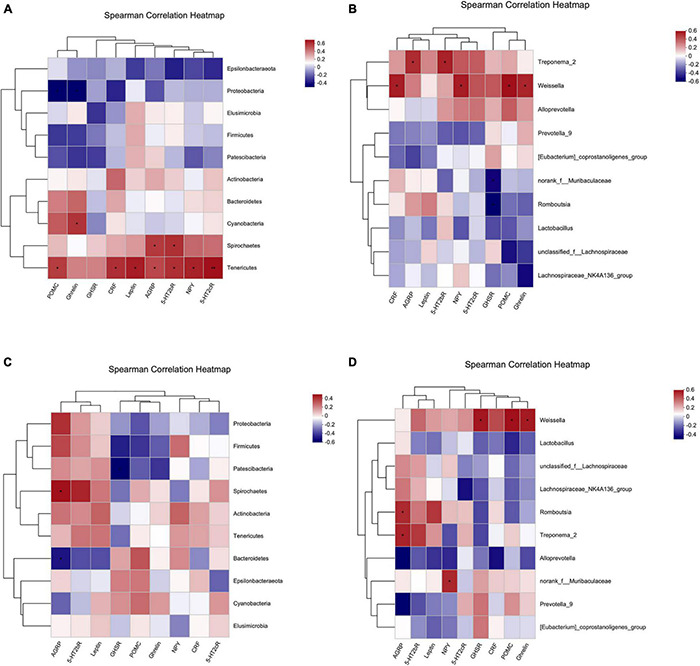
Spearman correlation heatmap. **(A,B)** Correlation heatmap of hypothalamic Ghrelin pathway-related molecules and intestinal dominant microbiota. **(C,D)** Correlation heatmap of gastric Ghrelin pathway-related molecules and intestinal dominant microbiota. **(A,C)** Phylum level. **(B,D)** Genus level. The *X* and *Y*-axes are Ghrelin pathway-related molecules and intestinal dominant microbiota, respectively, and the correlation *R*-value and *P*-value are obtained through calculation. The *R*-value is shown using different colors in the figure. If the *P*-value is less than 0.05, it is marked with *. The legend on the right is the color interval of different *R* values. The species and environmental factor cluster trees are presented on the left and upper side, respectively. *: 0.01 < *P* ≤ 0.05, **: 0.001 < *P* ≤ 0.01.

Figure 13A shows the correlation between the dominant intestinal strains at the phylum level and molecules in the hypothalamic Ghrelin pathway. *Tenericutes* had a significant positive correlation with *Pomc, Crf, Npy,5-Ht_2c_R, Leptin, Agrp* and *5-Ht_2b_R* (*0.01 < *P* ≤ 0.05, ^**^: 0.001 < *P* ≤ 0.01), *Spirochaetes* had a significant positive correlation with *Agrp* and *5-Ht_2c_R* (*: 0.01 < *P* ≤ 0.05), *Cyanobacteria* had a significant positive correlation with *Ghrelin* (*: 0.01 < *P* ≤ 0.05), and *Proteobacteria* had a significant negative correlation with *Pomc* and *Ghrelin* (*: 0.01 < *P* ≤ 0.05).

Figure 13B shows the correlation between the dominant intestinal strains at the genus level and molecules in the hypothalamic Ghrelin pathway. *Treponema_2* had significant positive correlation with *Agrp* and *5-Ht_2b_R* (*: 0.01 < *P* ≤ 0.05), *Weissella* had significant positive correlation with *Crf, Npy, Pomc* and *Ghrelin* (*: 0.01 < *P* ≤ 0.05), and *Norank_f_Muribaculaceae* and *Romboutsia* were negatively correlated with *Ghsr* (*: 0.01 < *P* ≤ 0.05).

Figure 13C shows the correlation between the dominant intestinal strains at the phylum level and gastric Ghrelin pathway-specific molecules. *Patescibacteria* was negatively correlated with *Ghsr* (*: 0.01 < *P* ≤ 0.05), *Spirochaetes* had a significant positive correlation with *Agrp* (*: 0.01 < *P* ≤ 0.05), and *Bacteroidetes* had a significant negative correlation with *Agrp* (*: 0.01 < *P* ≤ 0.05).

Figure 13D shows the correlation between dominant intestinal strains at the genus level and molecules in the Ghrelin pathway in the stomach. *Weissella* had a significant positive correlation with *Ghsr, Pomc* and *Ghrelin* (*: 0.01 < *P* ≤ 0.05), *Romboutsia* and *Treponema_2* had a significant positive correlation with *Agrp* (*: 0.01 < *P* ≤ 0.05), and *Norank_f_Muribaculaceae* had a significant positive correlation with *Npy* (*: 0.01 < *P* ≤ 0.05).

## Discussion

Non-alcoholic fatty liver disease is a worldwide health concern. Recent studies show that intestinal microbiota imbalances play a role in the occurrence and development of this disease. Regulating the intestinal microbiota has become a new option for treatment of non-alcoholic fatty liver. In addition, many studies show that because NAFLD risk correlates with higher BMI, interventions aimed at reducing body weight and feed intake can help reduce the incidence of NAFLD. Prior research indicated that 919 syrup has great potential for treatment of NAFLD (Chen et al., 2020). The current study assessed the safety of 919 syrup and its regulatory impact on the appetite-related Ghrelin pathway and intestinal microbiota in rats with NAFLD. The therapeutic mechanism of 919 syrup on NAFLD was studied from different perspectives, providing a new entry point to assess the effectiveness of 919 syrup as a NAFLD treatment.

**FIGURE 14:**
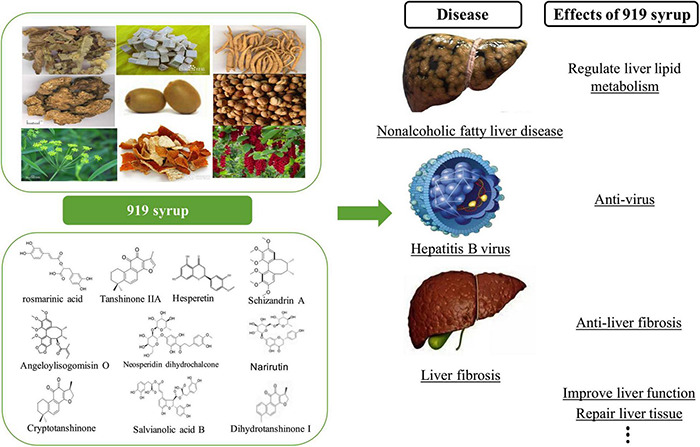
Previous experimental results of 919 syrup. The **upper left** of the figure shows the herbal compositions of 919 syrup, and the **lower left** of the figure shows the 10 main chemical components of 919 syrup. The **right side** of the figure shows the effects of 919 syrup in previous experiments.

In a prior study, the active components of 919 syrup were analyzed using HPLC/ESI-MS (Xu et al., 2017), and major chemical substances, including *rosmarinic acid, hesperitin, neosperidin dihydrochalcone, narirutin, schizandrin A, angeloylisogomisin O, salvianolic acid B, dihydrotanshinone I, tanshinone IIA*, and *cryptotanshinone* were found (Figure 14). These chemical components inhibited inflammation (Jin et al., 2014), oxidation (Petersen and Simmonds, 2003), fibrosis (Wat et al., 2016), and damage to liver function (Zhang et al., 2017). They also reduced degeneration and necrosis of liver cells (Hu et al., 2014) and regulated liver lipid metabolism (Jeong et al., 2019). The first part of the current study assessed the toxicity of 919 syrup and found no significant negative impact on the body weight, feed intake, blood glucose, or hepatorenal function of normal rats. In addition, 919 syrup had no obvious impact on liver histology, indicating that 919 syrup is a non-toxic Chinese herbal medicine mixture.

**FIGURE 15:**
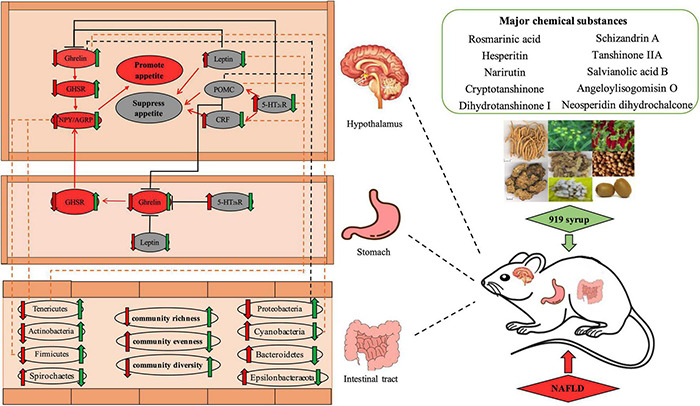
Effect of Chinese herbal medicine mixture 919 syrup on the regulation of the appetite-related Ghrelin pathway and intestinal microbiota in rats with non-alcoholic fatty liver disease. Red arrow (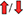): Effect of NAFLD on gene expression levels or microbiota abundance. Green arrow (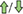): Effect of 919 syrup on gene expression levels or microbiota abundance. Upward arrow (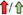): Promotion effect. Downward arrow (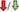): Inhibition effect. Solid red line (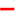): Promoting effect of the gene on the gene. Solid black line (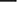): Inhibiting effect of the gene on the gene. Red oval box (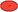): Appetite-promoting gene. Gray oval box (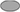): Appetite-suppressing gene. Dashed orange line (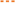): The positive correlation between intestinal bacteria and genes. Dashed black line (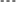): The negative correlation between intestinal bacteria and genes.

The appetite center is primarily located in the arcuate nucleus (ARC) and paraventricular nucleus (PVN) of the hypothalamus (Figure 15). ARC expresses CRF and POMC to suppress appetite and expresses NPY and AGRP to promote appetite. These two parts act together on the PVN, helping to regulate appetite. Ghrelin derived from gastric endocrine cells is a hunger hormone that increases appetite and feed intake (Diz-Chaves, 2011). It acts with Ghrelin synthesized in the hypothalamus on the ARC and PVN to regulate feed intake. When fat storage is excessive, more Leptin is secreted and acts on the Leptin receptor on ARC to regulate POMC, NPY and AGRP levels and reduce appetite and fat accumulation. Studies (Yakabi et al., 2010; Nahata et al., 2013) show that activation of 5-HT_2b_ receptors in the Ghrelin signaling pathway inhibit the secretion of acylated Ghrelin by X/A-like cells in the gastric mucosa, reducing Ghrelin binding to its receptor, GHSR, decreasing activation of NPY/AGRP in the hypothalamus of the appetite promotion system, and lowering appetite. In the central nervous system, the appetite suppression related gene, CRF/POMC, binds to the 5-HT_2C_ receptor, inhibiting secretion of Ghrelin in the blood. In addition, activation of the 5-HT_2C_ receptor in the hypothalamus inhibits secretion of acylated Ghrelin. The balance between anorexic f and appetite-stimulating factors plays a crucial role in the regulation of appetite. If this balance is broken, appetite disorders and metabolic diseases, such as obesity or severe malnutrition, can occur, which can lead to NAFLD.

In the second part of this study, the regulatory effect of 919 syrup on feed intake and body weight of NAFLD rats was studied by assessing the appetite-related Ghrelin pathway in the brain and stomach (Figure 15). Many studies (Fei et al., 2012; Li et al., 2017) show that abnormal weight and intake are closely related to the formation and development of NAFLD. Overweight and obesity are risk factors for NAFLD and hyperlipidemia, and metabolic syndrome and NAFLD are significantly higher in obese than non-obese people. Thus, interventions aimed at reducing body weight and feed intake can help improve lipid metabolism and reduce NAFLD (Xing et al., 2013). The results shown here indicate that 919 syrup could reverse the abnormal expression of Ghrelin pathway-related genes in the brain and stomach of NAFLD rats. Moreover, expression of *Npy*/*Agrp* in the Ghrelin pathway in the brain and stomach was consistent with the change in body weight. Increased expression of Ghrelin in the hypothalamus and stomach did not increase feed intake of rats, which may be related to Ghrelin resistance. These results indicated that 919 syrup could reverse the abnormal expression Ghrelin pathway-related genes in the brain and stomach of rats with NAFLD, and that the decrease in body weight and feed intake observed in treated rats might be related to regulation of *Npy*/*Agrp*.

More research indicates that gut microbes play an important role in the development of NAFLD. The total number and population structure of intestinal microbiota changes in response to various environmental, immune, and nutritional states (D’Argenio and Salvatore, 2015; Oh et al., 2016). Patients with NAFLD have different degrees of intestinal microbiota imbalance (Chassaing et al., 2014). Cotillard et al. (2013) and Le Chatelier et al. (2013) found that people with low microbial abundance had a higher risk of developing metabolic diseases than those with high microbial abundance. Low abundance of intestinal microbiota was associated with higher levels of serum triglycerides and free fatty acids, higher body fat content, and increased pro-inflammatory bacteria such as *Bacteroides* and active *Ruminococcus*, putting individuals at increased risk for NAFLD, diabetes and ischemic cardiovascular disease. It is shown that intestinal probiotics help to reduce serum triglycerides and cholesterol, liver fat accumulation and liver cell steatosis (Guo et al., 2011). The intestinal microbiota analysis conducted in this study showed that the diversity and richness of the intestinal microbiota in normal rats was significantly higher than that seen in NAFLD rats, consistent with results reported previously (Cotillard et al., 2013; Le Chatelier et al., 2013). In addition, 919 syrup reversed the decline in intestinal microbial community richness and diversity and increased intestinal microbial community uniformity in NAFLD rats, making the intestinal microbial community richness, diversity and evenness similar between the 919TJ and control groups (Figure 9). Compared to the control group, the structural proportions of intestinal microbiota in NAFLD rats changed significantly at both phylum and genus levels (Figures 10, 11). 919 syrup played an important role in the restoration of intestinal microbiota composition and structure induced by NAFLD (Figure 15). In addition, the proportion of *Lactobacillus* in the intestinal microbiota of rats in both the 919TJ and control groups were significantly higher than that seen in the NAFLD group, indicating that 919 syrup may contribute to the growth of intestinal probiotics.

Many studies show a connection between disturbance of the intestinal microbiota and the appetite of the host (Backhed et al., 2004; Turnbaugh et al., 2006; Schéle et al., 2013). Appetite regulating hormones are an important means for the central nervous system to regulate appetite. Most of these are secreted by enteroendocrine cells, so the intestinal microbiota is likely to regulate appetite through gastrointestinal hormones. One study reported that germ-free mice prefer a high-fat, high-carbohydrate diet after receiving intestinal microbiota transplantation with a lower microbial richness (Kong et al., 2013). Adding SL#3, a dietary supplement supplemented with a mixture of *Lactobacillus* strains, to the diet of mice decreased expression of appetite hormones, AGRP and NPY, in the hypothalamus (Yadav et al., 2013). Changes in the intestinal microbiota affect dietary preference and the expression of appetite-related genes. The heatmap showing results from this experiment also indicated that the intestinal microbiota is correlated with appetite related genes (Figure 13). 919 syrup can improve the intestinal microbiota and expression of genes in the appetite-related Ghrelin pathway, thus regulating the appetite and body weight of NAFLD rats. These intestinal dominant microbiotas correlate significantly with genes expressed by the appetite-related Ghrelin pathway (Figure 15). It is speculated that 919 syrup induces changes in appetite-related gene expression by regulating the intestinal microbiota of NAFLD rats, thereby changing appetite. Future experiments will explore the intestinal microbiota metabolites related to the development of NAFLD, and the effect of 919 syrup on these metabolites.

This study shows that 919 syrup has a positive effect on protecting health and may be a critical herbal remedy for NAFLD and other ailments, including chronic liver disease and chronic fatigue syndrome. 919 syrup has a high curative effect, no toxic side effects, is highly abundant and inexpensive. The development and application of Chinese herbal medicine can make full use of natural herbal resources, which has great social and economic benefits.

## Conclusion

This study showed that 919 syrup had no negative effects on body weight, feed intake, blood glucose, hepatorenal function, and liver histology of normal rats. 919 syrup reversed the abnormal gene expression of the Ghrelin pathway in the brain and stomach under NAFLD conditions, regulated feed intake and body weight, and improved intestinal microbiota disturbance in rats with NAFLD. In summary, 919 syrup may have therapeutic effects on NAFLD by regulating feed intake, body weight and the balance of intestinal microbiota, and is a promising therapeutic drug for treatment of NAFLD.

## Data Availability Statement

The data presented in the study are deposited in the NCBI SRA database, accession number: PRJNA781932.

## Ethics Statement

The animal study was reviewed and approved by Animal Care and Use Committee of Fudan University Public Health Clinical Center.

## Author Contributions

All authors have contributed to the study conception and design, commented on previous versions of the manuscript, and read and approved the final manuscript. MC, PG, JX, and DP performed the material preparation, data collection, and analysis. MC wrote the first draft of the manuscript.

## Conflict of Interest

The authors declare that the research was conducted in the absence of any commercial or financial relationships that could be construed as a potential conflict of interest.

## Publisher’s Note

All claims expressed in this article are solely those of the authors and do not necessarily represent those of their affiliated organizations, or those of the publisher, the editors and the reviewers. Any product that may be evaluated in this article, or claim that may be made by its manufacturer, is not guaranteed or endorsed by the publisher.

## Supplementary Material

Supplementary Figure S1 Community Heatmap analysis of intestinal microbial. **(A)** Phylum level. **(B)** Genus level. Z: Control group. G: NAFLD group. T: 919TJ group.

Supplementary Figure S2 Community circos analysis of intestinal microbial. **(A)** Phylum level (Others: <0.001). **(B)** Genus level (Others: <0.02).

Supplementary Figure S3 Hierarchical clustering tree of intestinal microbial (phylum level). Z: Control group. G: NAFLD group. T: 919TJ group.

Supplementary Table S1 Sequences of the primers used for qRT-PCR.
