# Supplementary figures and images for: Effect of Chinese Herbal Medicine Mixture 919 Syrup on Regulation of the Ghrelin Pathway and Intestinal Microbiota in Rats With Non-alcoholic Fatty Liver Disease
Source: Front Microbiol. 2021 Dec 24;12:793854. doi: 10.3389/fmicb.2021.793854 (PMC8740226; doi:10.3389/fmicb.2021.793854)

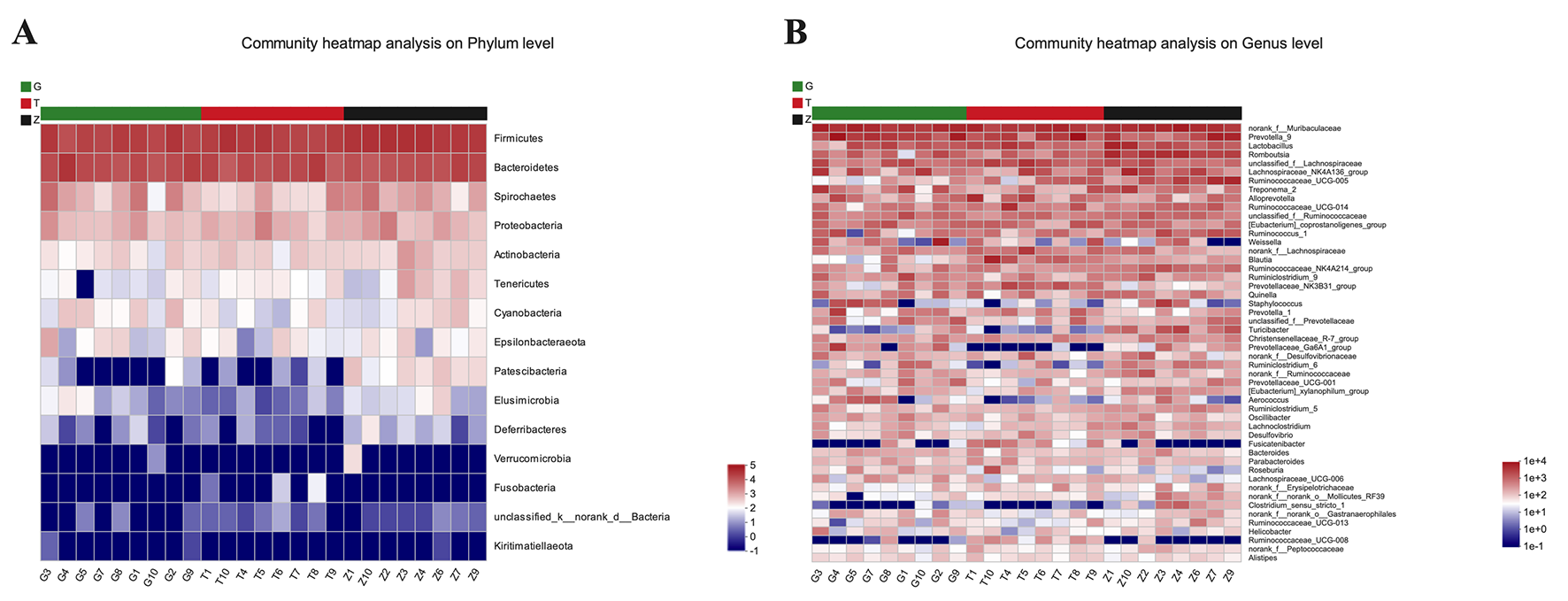

Supplement: Supplementary Figure S1 — Community Heatmap analysis of intestinal microbial. (A) Phylum level. (B) Genus level. Z: Control group. G: NAFLD group. T: 919TJ group. [file Image_1.TIFF]

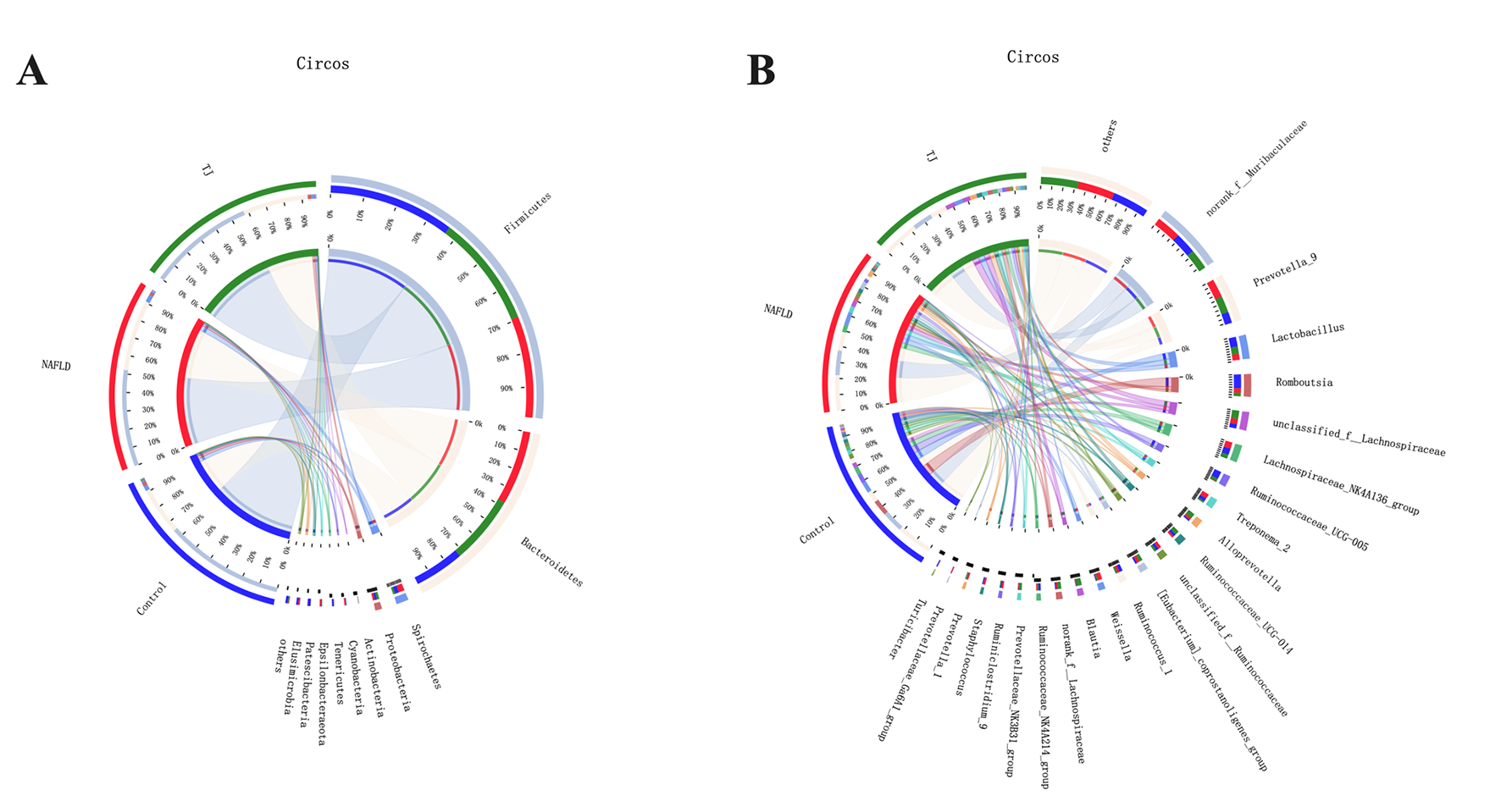

Supplement: Supplementary Figure S2 — Community circos analysis of intestinal microbial. (A) Phylum level (Others: <0.001). (B) Genus level (Others: <0.02). [file Image_2.TIFF]

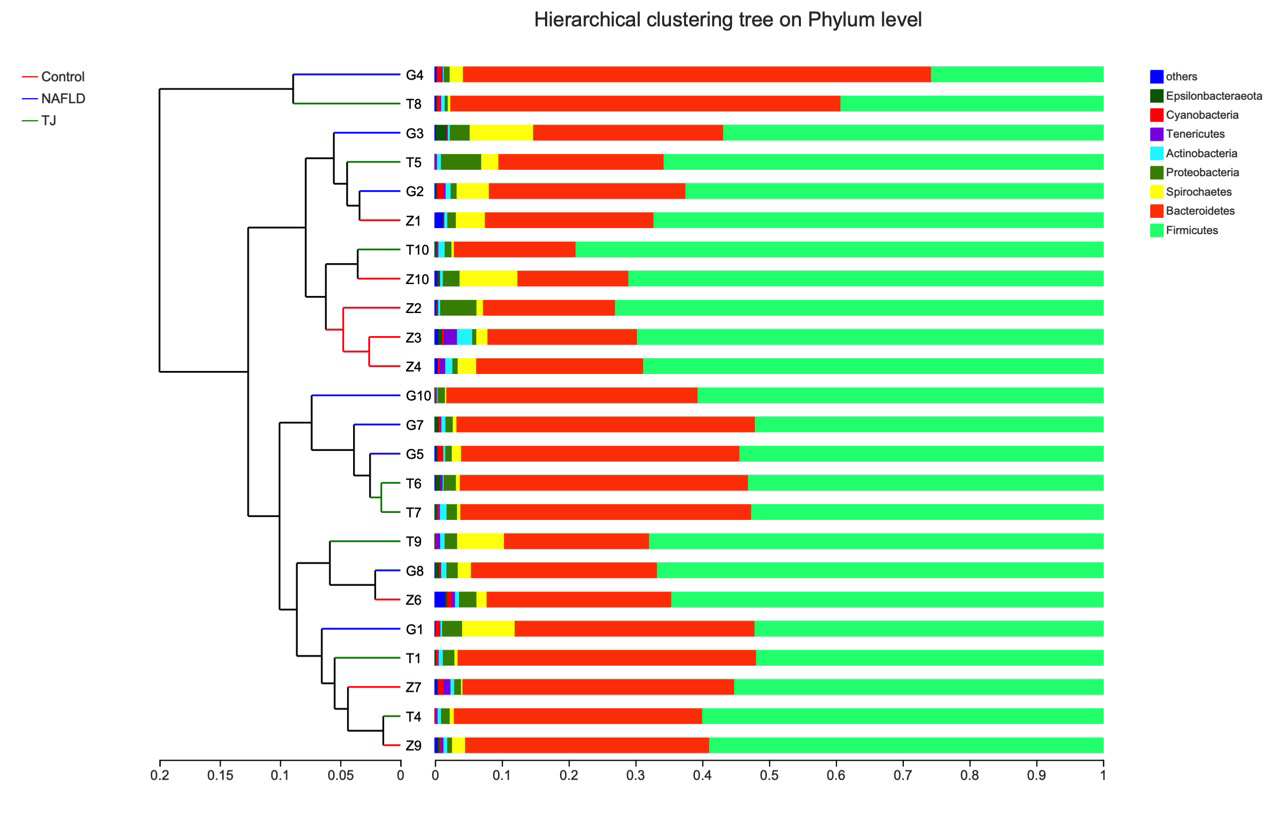

Supplement: Supplementary Figure S3 — Hierarchical clustering tree of intestinal microbial (phylum level). Z: Control group. G: NAFLD group. T: 919TJ group. [file Image_3.JPEG]
